# A Key ABA Catabolic Gene, *OsABA8ox3*, Is Involved in Drought Stress Resistance in Rice

**DOI:** 10.1371/journal.pone.0116646

**Published:** 2015-02-03

**Authors:** Shanlan Cai, Guobin Jiang, Nenghui Ye, Zhizhan Chu, Xuezhong Xu, Jianhua Zhang, Guohui Zhu

**Affiliations:** 1 College of Life Sciences, South China Agricultural University, Guangzhou, China; 2 Shenzhen Research Institute, The Chinese University of Hong Kong, Shenzhen, China; 3 State Key Laboratory of Agrobiotechnology, The Chinese University of Hong Kong, Hong Kong, China; 4 Guangdong Provincial Key Laboratory of Protein Function and Regulation in Agricultural Organisms, South China Agricultural University, Guangzhou, China; University of Missouri, UNITED STATES

## Abstract

Expressions of ABA biosynthesis genes and catabolism genes are generally co-regulated in plant development and responses to environmental stress. Up-regulation of *OsNCED3* gene, a key gene in ABA biosynthesis, has been suggested as a way to enhance plant drought resistance but little is known for the role of ABA catabolic genes during drought stress. In this study, we found that *OsABA8ox3* was the most highly expressed gene of the *OsABA8ox* family in rice leaves. Expression of *OsABA8ox3* was promptly induced by rehydration after PEG-mimic dehydration, a tendency opposite to the changes of ABA level. We therefore constructed rice *OsABA8ox3* silencing (RNA interference, RNAi) and overexpression plants. There were no obvious phenotype differences between the transgenic seedlings and wild type under normal condition. However, *OsABA8ox3* RNAi lines showed significant improvement in drought stress tolerance while the overexpression seedlings were hypersensitive to drought stress when compared with wild type in terms of plant survival rates after 10 days of unwatering. Enzyme activity analysis indicated that *OsABA8ox3* RNAi plants had higher superoxide dismutase (SOD) and catalase (CAT) activities and less malondialdehyde (MDA) content than those of wild type when the plants were exposed to dehydration treatment, indicating a better anti-oxidative stress capability and less membrane damage. DNA microarray and real-time PCR analysis under dehydration treatment revealed that expressions of a group of stress/drought-related genes, i.e. *LEA* genes, were enhanced with higher transcript levels in *OsABA8ox3* RNAi transgenic seedlings. We therefore conclude that that *OsABA8ox3* gene plays an important role in controlling ABA level and drought stress resistance in rice.

## Introduction

Drought is one of the most important environmental factors that restrict plant growth and seed production. Plants respond and adapt to the drought stress through various physiological and metabolic processes, including stomatal closure, repression of cell growth and photosynthesis, and activation of respiration, to increase the chance of survival [1, 2]. The molecular mechanism that regulates responses of plant to drought stress is extremely complicated, which involves expression change of thousands of genes in *Arabidopsis* [3]. Generally, signaling transduction in response to drought stress can be divided into two pathways: the ABA-dependent and ABA-independent pathway [1]. Most of the key genes in these pathways have been identified, such as transcription factors belonging to the class of DRE-binding protein (DREB)/C-repeat-binding factor (CBF), ABA-binding factor (ABF), MYC, and MYB [1, 4]. Although ABA has broad functions in plant growth and development, its main function is to regulate plant water balance and osmotic stress tolerance [5]. ABA accumulation is considered as an important mechanism in response to drought stress, which causes stomatal closure and induces expression of drought-related genes and consequently enhances plant tolerance to stress [6–8].

Plant endogenous ABA level is controlled by the balance of ABA biosynthesis and catabolism [9]. ABA is derived from carotenoid precursors and the cleavage of 9-*cis*-epoxycarotenoids to xanthoxin catalyzed by the enzyme 9-*cis*-epoxycarotenoid dioxygenase (NCED) is a key regulatory step for ABA biosynthesis [10, 11]. The hydroxylation of ABA to phaseic acid (PA) by ABA 8’-hydroxylase, a cytochrome P450 monooxygenase, is considered as the main ABA catabolic approach [7, 12]. Arabidopsis *CYP707A* genes are the firstly reported genes encoding ABA 8’-hydroxylase [13, 14] which are involved in several physiological processes, such as seed dormancy and germination, dehydration and rehydration, nitric oxide (NO) and sugar signal responses, and stomatal movement [15–18]. Rice ABA 8’-hydroxylase is encoded by three homologous genes *OsABA8ox1, OsABA8ox2* and *OsABA8ox3* [19]. Previous reports have partly described the functions of rice *OsABA8ox* genes. Expression of *OsABA8ox1* gene can be induced by ethylene which leads to the rapid decrease of ABA levels and consequently enhances elongation of submerged rice shoots [19, 20]. *OsABA8ox2* and *OsABA8ox3* are involved in glucose induced delay of seed germination [17]. Despite the fact that ABA catabolism plays important roles in many physiological processes, little has been deduced concerning the role of ABA catabolic genes in response to drought stress in rice. Here, we report that rice *OsABA8ox3* is a key gene regulating ABA accumulation under drought stress. Decreased *OsABA8ox3* expression by RNA interference enhanced rice drought resistance while overexpression of this gene showed hypersensitive response to drought stress.

## Materials and Methods

### Generation of RNA interference and overexpression plants

To construct the overexpression vector, the entire cDNA sequence of *OsABA8ox3* (NM_001069901) was PCR-amplified using the primers 5’CGGGGTACCTTTGGATGGCAGCCTCCTTCGTC3’ and 5’CGGACTAGTTTTCTCCCCGGACTTCCCTTGAG3’, then inserted into the pYL vector (a gift from Dr. Yao-Guang Liu, South China Agricultural University, Guangzhou) under the ubiquitin promoter. To generate the *OsABA8ox3* RNA interference plants, a 485 bp cDNA fragment was amplified with the primers 5’GAAGGATCCTACTCCCAAGACCCCAACGTCT3’ and 5’TCCCAAGCTTCTGTTGGGGAAGGAGTTGTAGC3’ and ligated into pYLRNAi vector, the inverse DNA fragment was amplified using the vector-specific primers and cloned into the same vector. The constructs were then introduced into rice (*Oryza sativa* L. cv. Zhonghua 11) by Agrobacterium- mediated transformation [21]. The transgenic rice plants that showed single insertion in T0 and 3:1 segregation ratios in the T1 were used in this study (S1 Fig.).

### Plant growth and stress treatment

Rice Zhonghua 11, pYL- transformed Zhonghua 11 (as WT), and transgenic seedlings were grown in a greenhouse at day/night temperature of 28/24°C with 14-h day and 10-h night periods. To mimic physiological dehydration experiment, the four-leaf-stage seedlings were grown in the Kimura B nutrient solution with 20% polyethylene glycol (PEG)-6000 for 4 h, and then transferred to Kimura B solution for rehydration. Samples were harvested at the referred time point to study the expression profiles of *OsABA8ox* gene family, ABA contents, antioxidant enzyme activities and DNA microarray. Soil drought stress was performed at five-leaf stage by stopping irrigation for 10 d and the drought-stressed seedlings were re-watered for 4 d.

### Gene expression analysis by quantitative real-time PCR (qRT-PCR)

Total RNA was extracted from rice seedlings using an RNA Easy Plant Mini Kit (Qiagen, CA) and then digested with DNase I (Amersham, USA) to eliminate genomic DNA contamination. First-strand cDNA was synthesized using a SuperScript first-strand synthesis system (Invitrogen, USA). Transcript levels of selected genes were measured by qRT-PCR using a iCycler (Bio-Rad, USA) with iQ SYBR Green Supermix (Bio-Rad, USA). The data was normalized to the amplification of rice *ACTIN2* gene. For each sample, the mean value from three qRT-PCR reactions was adapted to calculate the transcript abundance. Primer sequences used for qRT-PCR are listed in S1 Table, some of them were designed according to the previous publications [17, 22].

### DNA microarray analysis

The four-leaf stage leaves sampled at 0 h and 2 h after 20% PEG treatment from WT and *OsABA8ox3* RNAi-9 line were used for DNA microarray analysis. Total RNA was isolated using TRIzol reagent and purified by RNeasy spin columns (Qiagen, Germany). DNA microarray analysis was performed using Affymetrix Rice Genome Array by standard protocol (Affymetrix). GCOS software (Affymetrix Genechip Operating software) was used for data collection and normalization, and the values were log_2_ transformed. All the data about gene expression profiles were submitted to Gene Expression Omnibus, NCBI (Accession number GSE62308). The pathway analysis of the differential expressed genes between WT and RNAi-9 was performed using the MapMan software [23]. Partial up-regulated genes from GeneChip analysis were confirmed by real-time PCR.

### ABA contents, MDA contents and antioxidant enzyme assays

For estimation of endogenous ABA levels, 0.2 g seedlings were homogenized in 1 ml of distilled water and then shaken at 4°C overnight. The homogenates were centrifuged and the supernatant was directly used for ABA assay. ABA content was determined using the radioimmumoassay (RIA) method as described previously [17]. The MDA contents were measured according to the method of Heath and Packer [24]. The results are expressed as mg g^−1^ FW of the seedlings. For estimation of the antioxidant enzyme activities, 0.2 g of each fresh sample was homogenized in 5 ml of 50 mM chilled phosphate buffer (pH 7.0) containing 1 mM EDTA and 1% polyvinylpyrrolidone. The homogenate was centrifuged at 12 000 g for 20 min at 4°C and the supernatant was used for enzyme assays. The superoxide dismutase (SOD) activity was estimated by monitoring inhibition of the photochemical reduction of nitro blue tetrazolium (NBT) according to the method of Giannopolitis and Ries [25]. Catalase (CAT) activity was assayed from the rate of H_2_O_2_ decomposition as measured by decrease of absorbance at 240 nm, following the procedure of Aebi [26]. Peroxidase (POD) activity was assayed according to Chance and Maehly [27], and the activity was determined by monitoring the increase of absorbance at 470 nm.

### Seed germination experiment

The WT, *OsABA8ox3* RNAi- and overexpression- transgenic seeds were surface sterilized and directly sown on the sterile filter papers containing 0, 1, and 5μM ABA for seed germination assay. Seeds were placed in a growth chamber with 12 h light and 12 h dark at 28°C to facilitate germination. Germination ratio (based on radicles beyond 1 mm) was recorded after 4 d imbibition. Seeds were sown on the 1/2 MS medium for root length assay. Each plate contained 40 seeds. Every experiment was repeated three times.

## Results

### Rice *OsABA8ox3* is a drought responsive gene

**Figure 1:**
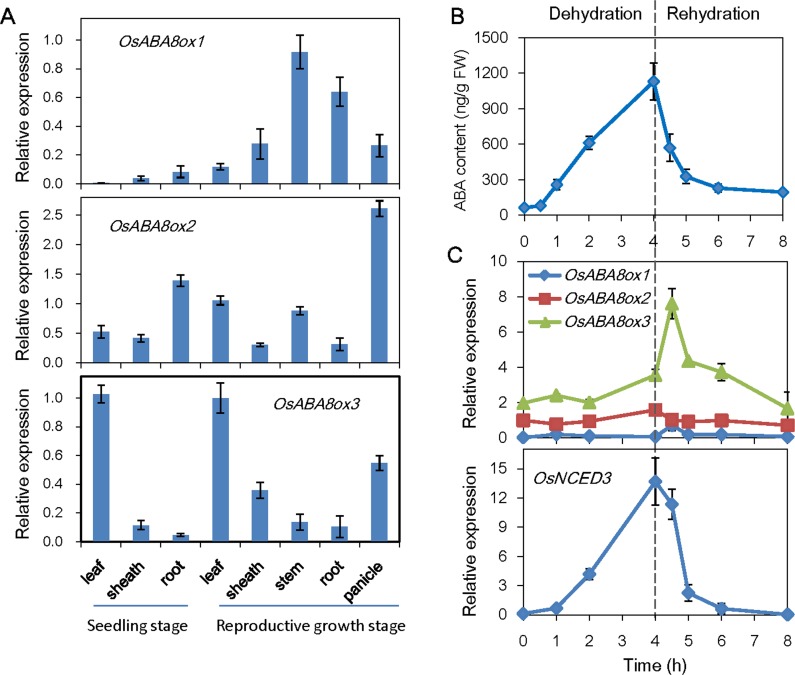
ABA accumulation and expression analysis of the *OsABA8ox* genes. (A) Expression patterns of *OsABA8ox* genes in different tissues of rice. (B) ABA accumulation and (C) *OsABA8ox* genes expression in rice leaves during PEG-mimic dehydration treatment and rehydration. Four-leaf stage seedlings were treated with 20% PEG for 4 h, and then transferred to nutrient solution for rehydration. Error bars are standard deviations based on three replicates.

Abscisic acid (ABA) is a well characterized drought responsive phytohormone, which plays important roles in adapting plants to environmental stress [28]. Dynamic change of ABA content depends on both ABA biosynthesis and its catabolism [29]. To test which ABA catabolic genes are responsible for the ABA accumulation subjecting to drought stress, the expression patterns in various tissues and expression variations under PEG treatment of three homologous genes, *OsABA8ox1, OsABA8ox2* and *OsABA8ox3*, were quantitative analyzed using qRT-PCR. As shown in Fig. 1A, expression of *OsABA8ox1* was hardly to be detected in the seedlings and relative higher in stems and roots at the reproductive stage. *OsABA8ox2* gene was preferentially expressed in roots at the seedling stage and panicles at the reproductive stage. *OsABA8ox3* gene was mainly expressed in leaves at both seedling stage and reproductive stage, whereas was lowly expressed in roots (Fig. 1A). From Fig. 1B, ABA was quickly accumulated in the four-leaf stage leaves when treated with 20% PEG and reached almost 1.2 μg per gram of fresh weight after 4 h treatment. Conversely, ABA content was rapidly reduced and recovered to the basal level after rehydration (Fig. 1B). Expression of ABA catabolic genes showed no obvious variation during dehydration, except for slight up-regulation of *OsABA8ox3* gene, which may be resulted from the feed-back regulation by the increasing of ABA content and *OsNCED3* expression (Fig. 1C). *OsABA8ox3* expression was promptly induced by rehydration, which coupling with the decrease of *OsNCED3* expression, responsible for the rapid decreasing of ABA level in rice leaves (Fig. 1B, C).

### *OsABA8ox3*-RNAi plants improve rice drought tolerance

**Figure 2:**
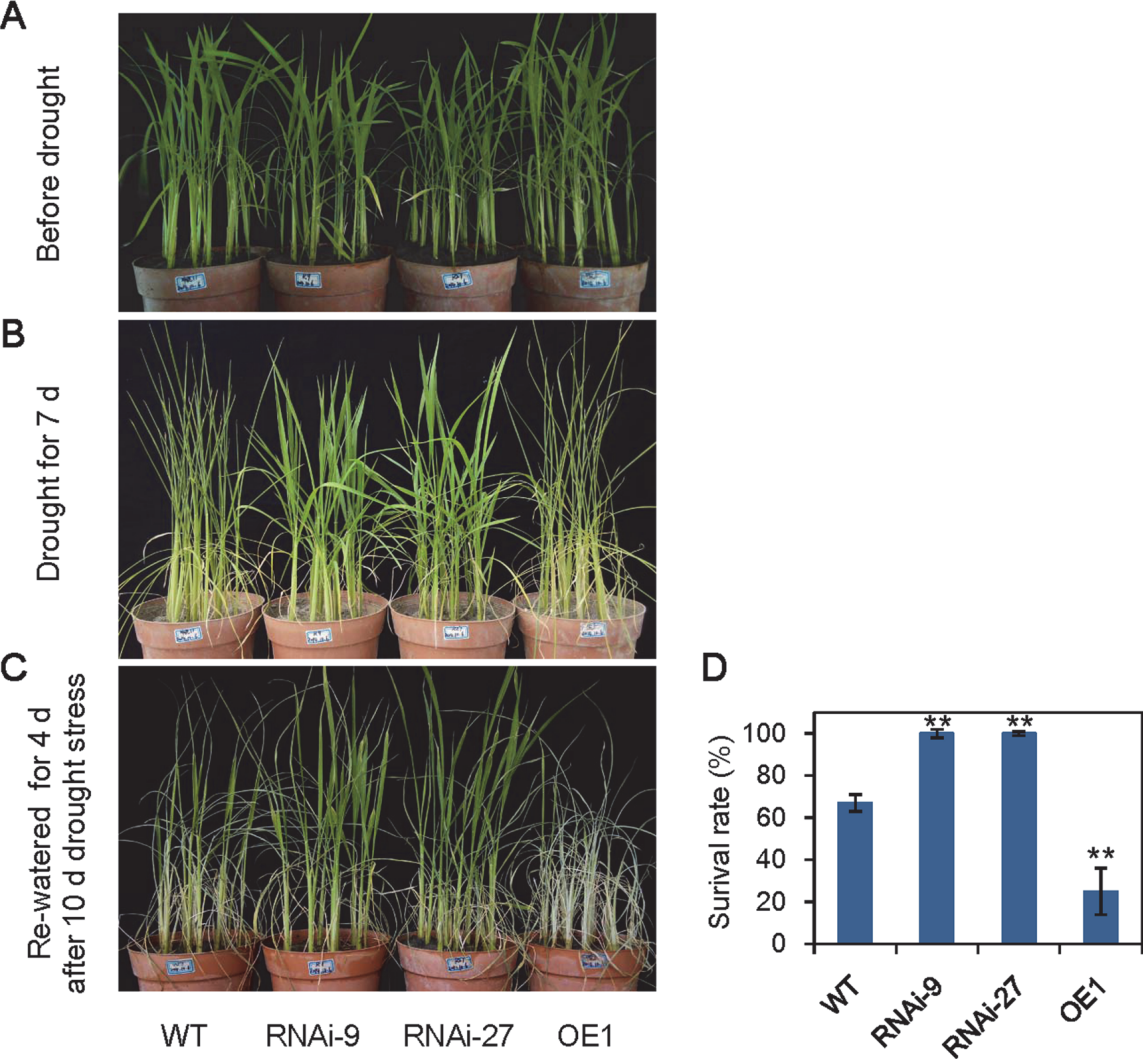
Stress tolerance assays of wild type plants and the transgenic rice. (A) Relative expressions of *OsABA8ox3* gene in RNAi and overexpression lines; (B) Performance of WT control and *OsABA8ox3* RNAi-9, RNAi -27 and overexpression lines after 7 d soil drought stress and 4 d recovery. (C) Survival rate of rice seedlings after drought stress and rewatering. Error bars are standard deviations based on three replicates (n > 40). ** Indicates significant difference from wild-type at P = 0.01.

To confirm the role of *OsABA8ox3* gene in drought stress, *OsABA8ox3* RNA interference rice plants were produced with a 485 bp Open Reading Frame (ORF) region as the targeted interference DNA. We totally got 32 transgenic T0 lines. To get the homozygous lines, the T1 seedlings were further screened by 0.1% hygromycin solution, and only 4 lines which showed the segregation ratios as 3:1were obtained. To confirm the results, the T0 leaves of these 4 lines were Southern blotted using the *HPT* gene as probe. As shown in S1 Fig., all the 4 RNAi lines showed single copy genotypes (S1A Fig.). Two independent RNAi lines (RNAi-9 and RNAi-27) which showed significantly decreased expression of *OsABA8ox3* gene were used in this study (S1B Fig.). As shown in Fig. 2, the two *OsABA8ox3*-RNAi lines significantly improved rice resistance to drought stress compared with WT. After 7 days of soil drought stress under natural condition, the whole plants of WT leaves showed severe wilting phenotype, while these phenotypes appeared only at the old leaves or leaf apex in the *OsABA8ox3*-RNAi lines (Fig. 2B). The plants were re-watered after total 10 days of soil drought treatment. From Fig. 2C, almost all the RNAi lines recovered to vigorous after 4 days of rehydration, while only about 70% WT seedlings were survived (Fig. 2C, D). The experiment was also carried out by using 20% PEG treatment which showed similar results as soil drought treatment (S2 Fig.).

We also generated *OsABA8ox3* overexpression transgenic plants by constructing the full length of *OsABA8ox3* gene under the control of the ubiquitin promoter. 11 transgenic T0 lines were acquired and only one homozygous line (OE1) was selected for further study after screening by the above referred method (S1 Fig.). As shown in Fig. 2, *OsABA8ox3* overexpression seedlings showed much more sensitivity to soil drought stress compared with WT. The survival rate of *OsABA8ox3* overexpression line was only 20–30% after re-watering (Fig. 2). Taken together, these results suggested that *OsABA8ox3* plays crucial roles in drought resistance in rice.

### *OsABA8ox3*-RNAi plants enhance ABA content and improve antioxidant enzyme activities under drought stress

**Figure 3:**
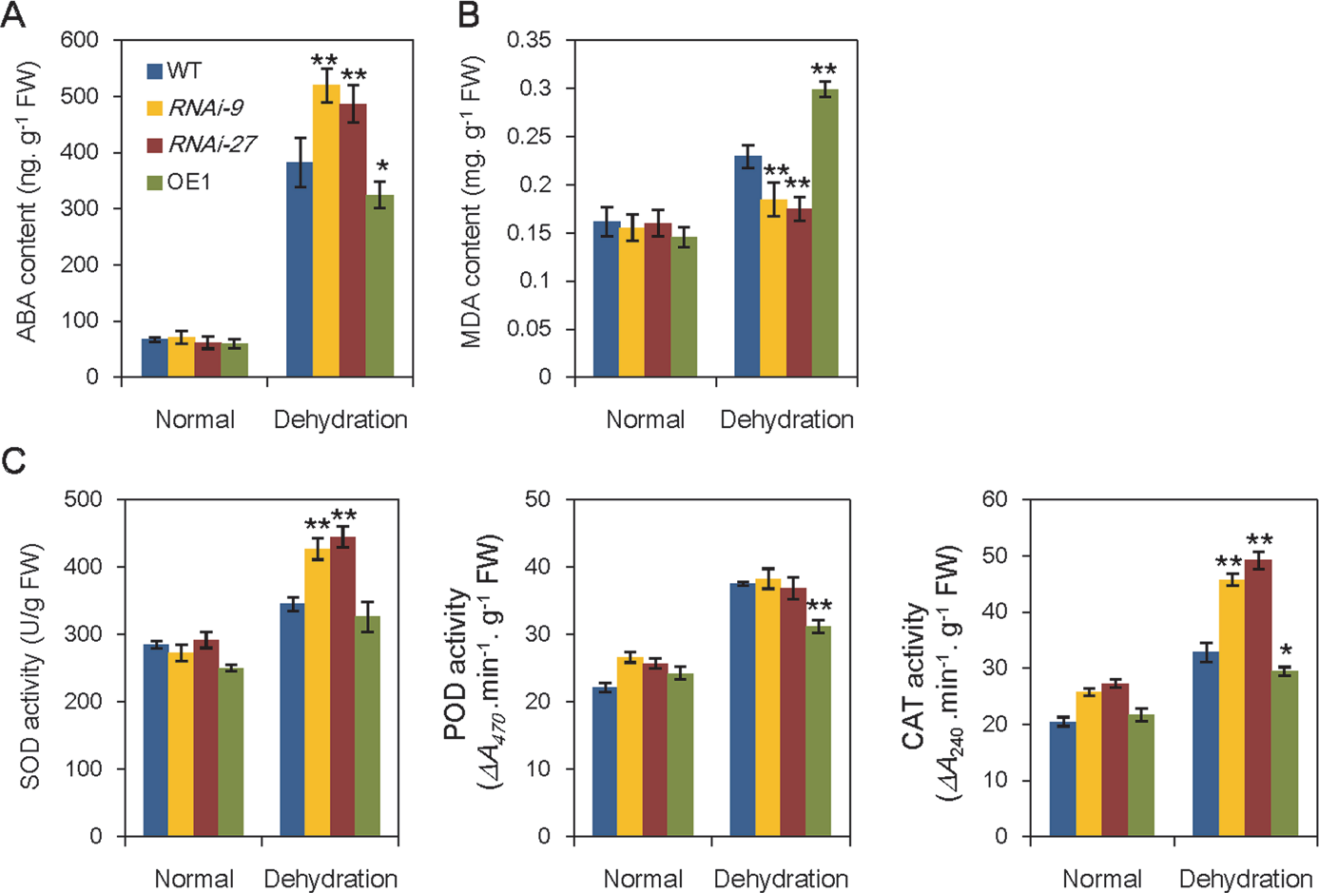
Physiological changes in the *OsABA8ox3*-RNAi and -overexpression transgenic plants. (A) ABA contents, (B) MDA contents and (C) antioxidant enzyme activities in the WT and *OsABA8ox3*-RNAi and -overexpression transgenic seedlings under normal condition and PEG treatment. Four-leaf stage seedlings were treated with 20% PEG for 2 h, then the samples were quickly frozen with liquid nitrogen. Error bars are standard deviations based on three replicates. ** Indicates significant difference from wild-type at P = 0.01.

As *OsABA8ox3*-RNAi plants showed enhanced drought tolerance, we examined the ABA content and several antioxidant enzyme activities under drought stress at the seedling stage. ABA content in both WT and *OsABA8ox3*-RNAi plants was increased after 2 h of PEG-mimic dehydration treatment. In comparison to the WT, *OsABA8ox3*-RNAi plants had higher ABA level (Fig. 3A), which may owe to the reduced expression of *OsABA8ox3* gene. Conversely, ABA content in *OsABA8ox3*-OE1 seedlings was comparably lower than that of WT in response to drought stress (Fig. 3A).

We further analyzed the Malondialdehyde (MDA) content as which is often used as a biomarker to measure cell membrane injury and cell and tissue oxidative damage [30]. The MDA content was significantly increased in WT seedlings after drought stress, the MDA increases were less in the *OsABA8ox3*-RNAi leaves, while much higher in the *OsABA8ox3*-OE1 leaves than that of WT (Fig. 3B), indicating that Inhibition of *OsABA8ox3* expression may reduce the oxidative damage under drought stress. We also determined the activities of several antioxidant enzymes, i.e. superoxide dismutase (SOD), peroxidase (POD), and catalase (CAT). As shown in Fig. 3C, drought stress-induced SOD, POD and CAT activities in the *OsABA8ox3*-RNAi seedlings was significantly higher than those in the WT seedlings, especially SOD and CAT. In contrast, POD and CAT activities were decreased in the *OsABA8ox3*-OE seedlings. These results suggested that higher antioxidant enzyme activity plays an important role in *OsABA8ox3*-RNAi plants under drought stress.

### *OsABA8ox3*-RNAi plants enhance expressions of stress responsive genes under drought stress

**Figure 4:**
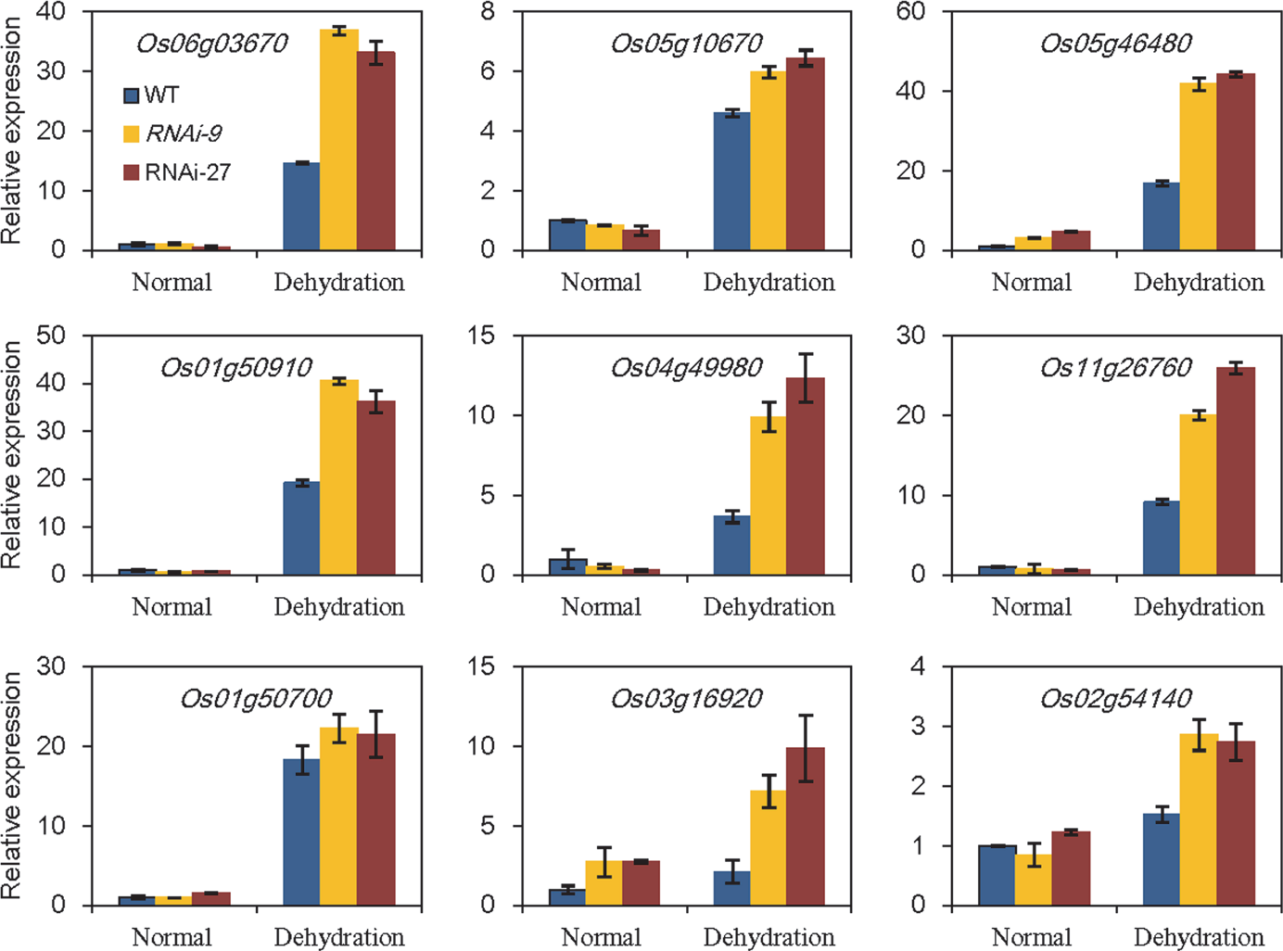
Relative expression levels of 9 stress-related genes in the WT and *OsABA8ox3* RNAi transgenic plants under normal and PEG-treated conditions detected by qRT-PCR. Four-leaf stage seedlings were treated with 20% PEG for 2 h, and then the samples were quickly frozen with liquid nitrogen for RNA extraction. The genes include dehydration-responsive element-binding protein (Os06g03670), zinc finger protein (Os05g10670), late embryogenesis abundant proteins (Os05g46480, Os01g50910 and Os04g49980), dehydrin family proteins (Os11g26760 and Os01g50700) and heat shock protein (Os03g16920 and Os02g54140).

To understand the molecular mechanism of drought stress resistance mediated by *OsABA8ox3* gene, we further checked the genome-wide expression profile changes in the *OsABA8ox3* RNAi-9 and WT seedlings using the Affymetrix GeneChip under the normal condition and drought stress. A total of 1436 genes showed greater than 2-fold higher expression levels in both WT and RNAi-9 seedlings after drought stress, and most of them had higher up-regulated folds in RNAi-9 seedlings than that of WT (S2 Table). Noticeably, many of these genes have been annotated to be involved in stress response, hormone metabolism, signal transduction and basic metabolisms (S3 Table). Several of these genes were further validated by qRT-PCR, such as late embryogenesis abundant (LEA) proteins (Os05g46480, Os01g50910 and Os04g49980), dehydrin family proteins (Os11g26760 and Os01g50700), heat shock protein (Os03g16920 and Os02g54140), dehydration-responsive element-binding (DREB1A) protein (Os06g03670) and zinc finger protein (Os05g10670). From Fig. 4, all of the selected genes showed enhanced expression levels after drought stress and, moreover, their expression levels in *OsABA8ox3* RNAi-9 and RNAi-27 seedlings were significant higher than in the WT. These results confirmed the microarray data although the absolute values of the fold changes showed slight variation for some genes (Fig. 4, S4 Table).

### Increase in ABA sensitivity of *OsABA8ox3*-RNAi seeds during germination

**Figure 5:**
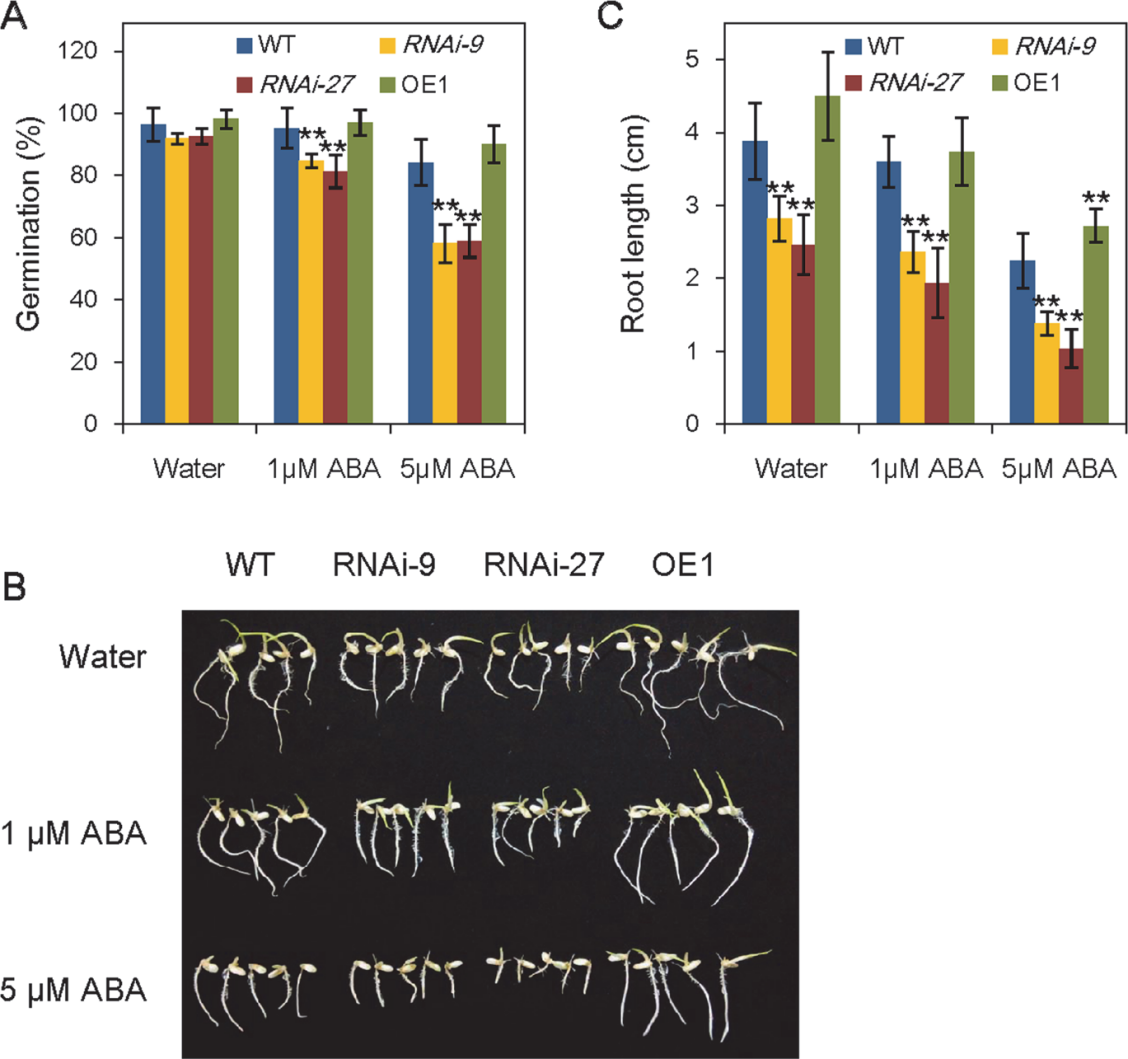
ABA sensitivity of the *OsABA8ox3* transgenic seeds during germination and post-germination growth. (A) Seed germination, (B) Post-germination root growth and (C) Root length of WT and *OsABA8ox3*-RNAi and -overexpression transgenic plants in response to exogenous ABA. Seeds treated with water and ABA (1μM and 5μM) were imbibed at 28°C to facilitate germination and post-germination growth. Error bars are standard deviations based on three replicates. ** Indicates significant difference from wild-type at P = 0.01.

We also checked the seed germination of *OsABA8ox3* transgenic plants since this physiological process is closely related to endogenous ABA level. The germination rates of WT, *OsABA8ox3* RNAi lines and overexpression line were compared under normal condition and ABA treatment. As shown in Fig. 5A, there were only slight reductions of germination rates in *OsABA8ox3* RNAi-9 and RNAi-27 seeds compared with that of WT in the normal medium, while the *OsABA8ox3* RNAi seeds seem much more sensitive than WT to exogenous ABA. For example, the seed germination rates reduced 11.0% and 14.6% in *OsABA8ox3* RNAi-9 and RNAi-27 seeds, respectively, compared with WT under 1 μM ABA treatment, as well as 30.9% and 30.0%, respectively, under 5 μM ABA treatment after 4 d imbibition (Fig. 5A). Similar results can also be observed by assaying the root length of WT and *OsABA8ox3* RNAi lines after 7 d growth on the 1/2 MS medium (Fig. 5B, C). The primary root growth of the *OsABA8ox3* RNAi-9 and RNAi-27 seedlings were obviously retarded compared with WT, either in normal condition or external supplying of ABA (Fig. 5B, C). In contrast, *OsABA8ox3* OE1 line slightly enhanced the seed germination rate and root length, especially under the condition of 5 μM ABA treatment (Fig. 5). The results suggested that the inhibition of *OsABA8ox3* gene expression in *OsABA8ox3*-RNAi plants showed hypersensitivity to exogenous ABA during seed germination and post-germination growth.

## Discussion

Plant endogenous ABA level is controlled by the equilibrium between ABA biosynthesis and catabolism [9]. In many cases, expressions of ABA biosynthesis genes and ABA catabolic genes were co-regulated in plant development and exogenous environmental stress. For example, ABA levels in tomato ovaries are regulated by *LeNCED1* and *SlCYP707A1* [31]. Controlling anther ABA homeostasis by regulating expression of *TaNCED1, TaNCED2* and *TaABA8′OH1* in wheat is important for reproductive stage stress tolerance [32]. Both of the ABA biosynthesis gene *AtNCED3* and ABA catabolic gene *AtCYP707A3* are well characterized to be the key genes in response to drought stress in *Arabidopsis* [11, 33]. Hwang et al [34] proved that rice *OsNCED3* is involved in ABA biosynthesis and drought tolerance through ectopic expression in *Arabidopsis*. Our data also showed that *OsNCED3* is crucial for rice adapting to drought stress, and the down-regulation of *OsNCED3* in transgenic seedlings by RNA interference showed hypersensitivity to drought stress (data not shown). Despite the fact that ABA catabolism plays important roles in many physiological processes, little has been deduced concerning the role of ABA catabolic genes in drought stress in rice.

Expression profiles of ABA hydroxylase gene family *OsABA8ox1, OsABA8ox2* and *OsABA8ox3*, were quantitative analyzed in response to PEG-mimic drought stress. PEG has been widely used to mimic water stress for many cases, i.e. two common stress signaling pathways, the ABA dependent and ABA independent pathway, which have became a paradigm in plant stress biology, were revealed by PEG-mimic drought stress studies [35, 36]. From Fig. 1A, *OsABA8ox3* gene was of the highest expression level among the three *OsABA8ox* genes in rice leaves. Moreover, expression of *OsABA8ox3* was promptly induced by rehydration, which showed a contrary tendency with the changes of ABA level (Fig. 1B, C), implying *OsABA8ox3* may be the major ABA catabolic gene that negatively controls ABA level during drought stress. We therefore constructed rice *OsABA8ox3* RNAi and overexpression transgenic plants. The data showed that *OsABA8ox3* RNAi lines had enhanced drought resistance while the overexpression seedlings were hypersensitive to drought stress (Fig. 2). Accumulation of the endogenous ABA contents in *OsABA8ox3* RNAi-9 and RNAi-27 seedlings was higher than that of WT to confer drought stress (Fig. 3), indicating that inhibition of *OsABA8ox3* expression is responsible for the drought-induced ABA accumulation, which subsequently leads to higher drought resistance.

Considered as a stress hormone, ABA in plants is dramatically induced by water stress. In the past two decades, crosstalk between ABA signal and ROS signal has been intensively studied [37, 38], and it is now well known that drought induced-ABA plays a key role in triggering the increased generation of ROS [39, 40]. To protect cells from oxidative damage by over-accumulated ROS, the plants have evolved a complicated antioxidant defense system which is readily induced by increased ROS [41, 42]. Thus, the increased ABA levels in *OsABA8ox3* RNAi leaves under drought stress may stimulate antioxidant system and induce the expressions of ABA/drought- responsive genes. We therefore checked MDA content and activities of several typical antioxidant enzymes. The increase of MDA content, as a biomarker of cell membrane injury, was relatively lower in the *OsABA8ox3* RNAi plants than that of WT under drought stress (Fig. 3B). To copy with the overproduction of ROS, plants have developed numerous scavenging enzymes such as SOD, POD and CAT to adjust ROS homeostasis [41, 42]. In this study, *OsABA8ox3* RNAi plants had higher SOD and CAT activities, while *OsABA8ox3*-overexpression transgenic plants had lower POD and CAT activities compared with WT (Fig. 3C). We also checked the differential expression of genes between *OsABA8ox3*-RNAi line and WT seedling under the normal condition and drought stress, and noticed that more than 10 genes encoding LEA, dehydrin and HSP protein had higher up-regulated folds in *OsABA8ox3*-RNAi seedlings than that of WT (Fig. 4, S2 Table, S3 Table). LEA and dehydrin family proteins, which are found in seeds and vegetative organs of plants, have been reported to be associated with increased stress tolerance in plants [22, 43]. Up-regulation of these genes in the *OsABA8ox3*-RNAi plants might contribute to the resistance to drought stress.

ABA is one of the most important hormones of seed dormancy and germination. The decrease of ABA level and increase of gibberellins (GA) level are the essential prerequisite for seed germination [44, 45]. In an earlier study, we demonstrated that *OsABA8ox2* and *OsABA8ox3* are the key genes controlling ABA levels during seed germination in rice [17]. In this study, seed germination and primary root growth of the *OsABA8ox3*-RNAi lines showed hypersensitivity to exogenous ABA, while *OsABA8ox3*-overexpression transgenic plants decreased the sensitivity compared with WT (Fig. 5). These findings further confirmed that *OsABA8ox3* gene, as the ABA catabolic key gene, responses not only to drought stress, but also to seed germination and post-germination growth.

## Supporting Information

S1 Fig Screening of the *OsABA8ox3* transgenic lines. (A) Southern blotting of the transgenic lines. (B) Expression levels of *ABA8ox3* in WT, RNAi lines and overexpression lines analyzed by qRT-PCR. Transgenic lines RNAi9, RNAi27 and OE1 were selected for this study.
(TIF)

S2 Fig Stress tolerance assays of the wild type plants and the *OsABA8ox3* transgenic rice under 20% PEG treatment. (TIF)

S1 Table qRT-PCR primers in this study. (XLSX)

S2 Table Expression profiles of 2-fold up- and down-regulated genes in both WT and RNAi-9 transgenic seedlings after drought stress. (XLSX)

S3 Table The pathway analysis of the genes with higher up-regulated expression levels in RNAi-9 transgenic seedlings than WT after drought stress using the MapMan software. (XLSX)

S4 Table The expression fold changes of the selected genes for qRT-PCR analysis in microarray. (XLSX)
