# Supplementary figures and images for: A Key ABA Catabolic Gene, OsABA8ox3, Is Involved in Drought Stress Resistance in Rice
Source: PLoS One. 2015 Feb 3;10(2):e0116646. doi: 10.1371/journal.pone.0116646 (PMC4315402; doi:10.1371/journal.pone.0116646)

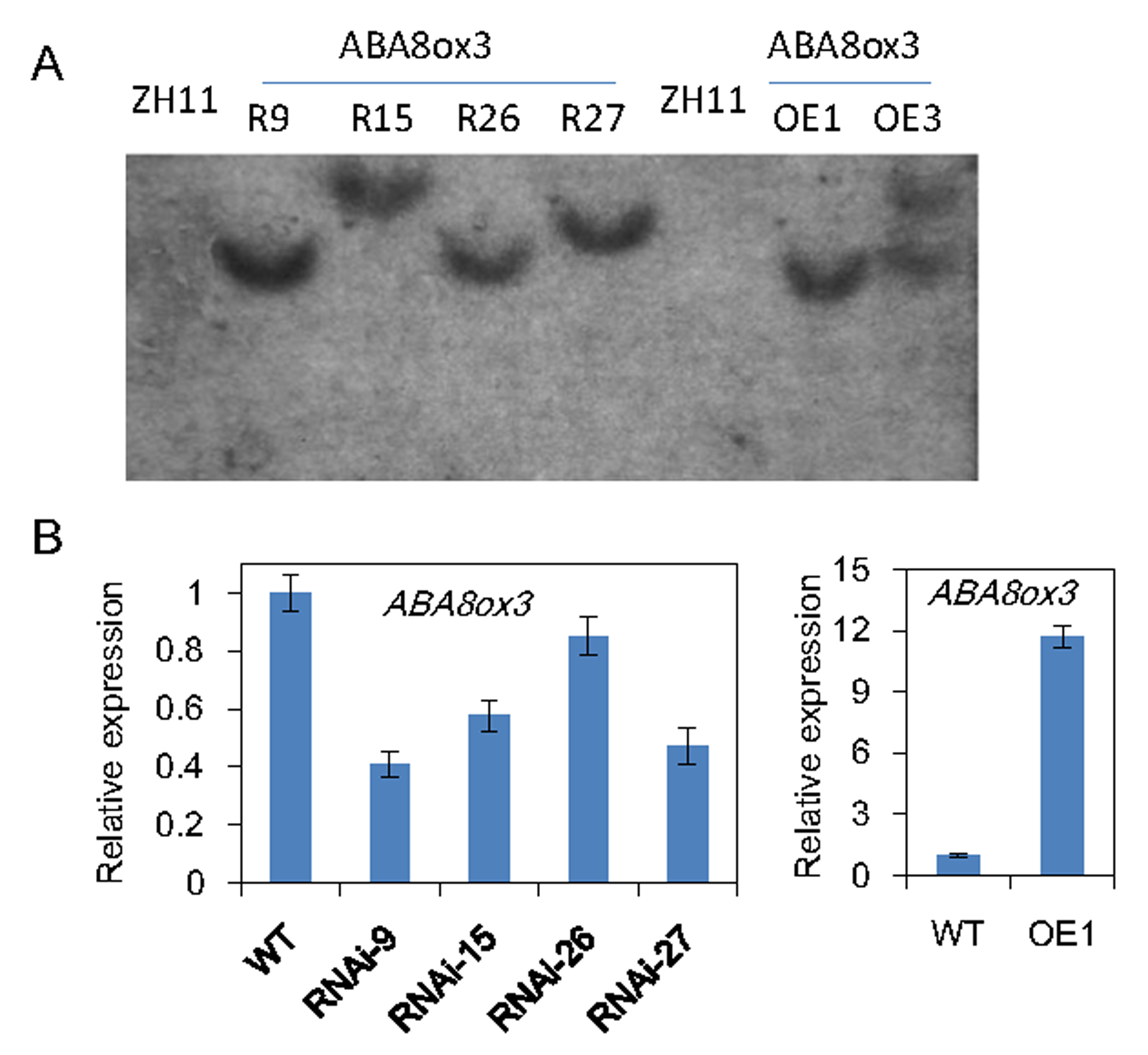

Supplement: S1 Fig — (A) Southern blotting of the transgenic lines. (B) Expression levels of ABA8ox3 in WT, RNAi lines and overexpression lines analyzed by qRT-PCR. Transgenic lines RNAi9, RNAi27 and OE1 were selected for this study. (TIF) [file pone.0116646.s001.tif]

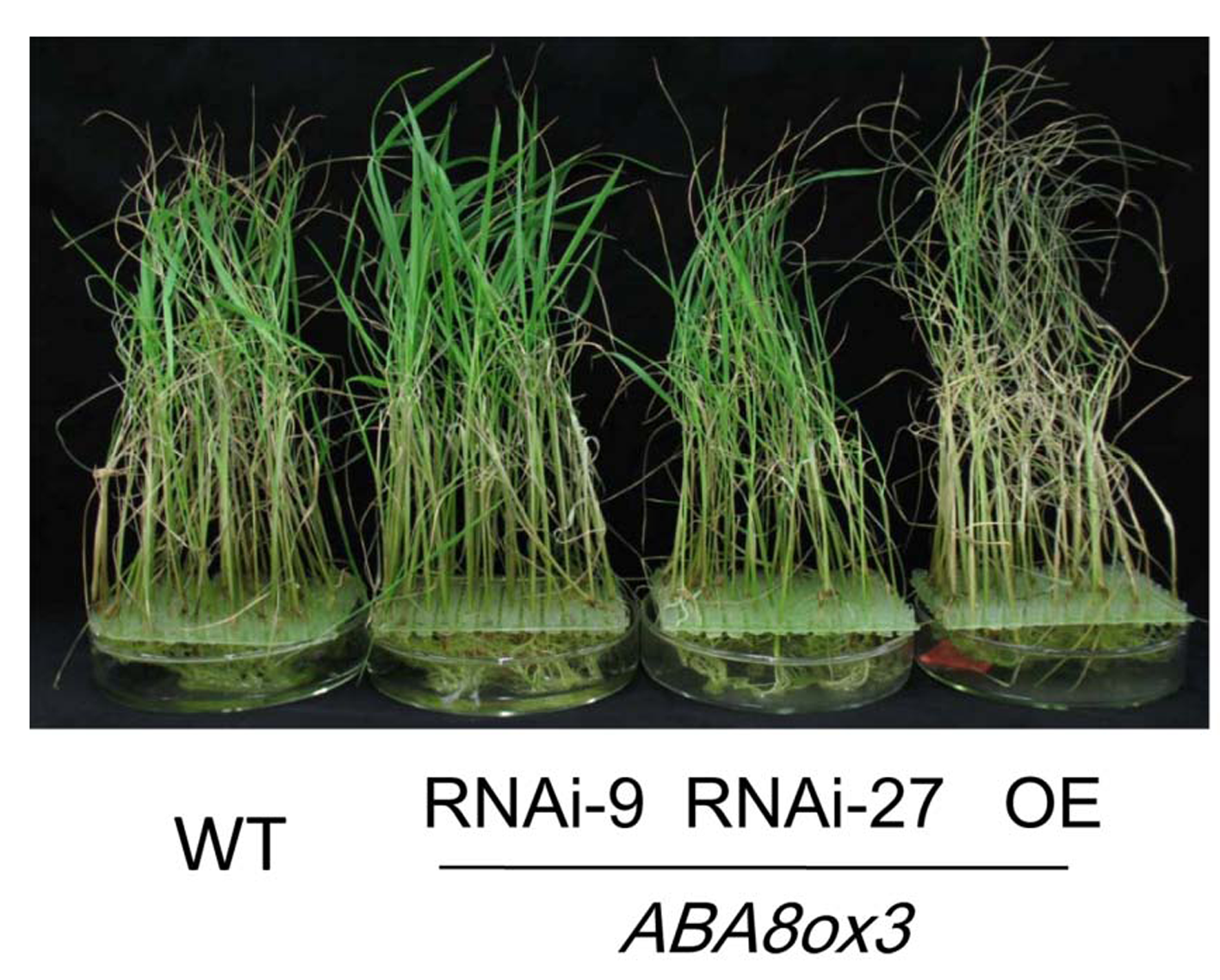

Supplement: S2 Fig — (TIF) [file pone.0116646.s002.tif]
